# Genome-wide characterization and expression profiling of HD-Zip gene family related to abiotic stress in cassava

**DOI:** 10.1371/journal.pone.0173043

**Published:** 2017-03-01

**Authors:** Zehong Ding, Lili Fu, Yan Yan, Weiwei Tie, Zhiqiang Xia, Wenquan Wang, Ming Peng, Wei Hu, Jiaming Zhang

**Affiliations:** Key Laboratory of Biology and Genetic Resources of Tropical Crops, Institute of Tropical Bioscience and Biotechnology, Chinese Academy of Tropical Agricultural Sciences, Haikou, Hainan, China; Institute of Genetics and Developmental Biology Chinese Academy of Sciences, CHINA

## Abstract

Homeodomain-leucine zipper (HD-Zip) gene family plays important roles in various abiotic stresses and hormone signaling in plants. However, no information is currently available regarding this family in cassava (*Manihot esculenta*), an important drought-tolerant crop in tropical and sub-tropical areas. Here, 57 *HD-Zip* genes (*MeHDZ01-57*) were identified in the cassava genome, and they were classified into four subfamilies based on phylogenetic analysis, which was further supported by their gene structure and conserved motif characteristics. Of which five gene pairs were involved in segmental duplication but none for tandem duplication, suggesting that segmental duplication was the main cause for the expansion of *MeHDZ* gene family in cassava. Global expression profiles revealed that *MeHDZ* genes were constitutively expressed, or not expressed, or tissue-specific expressed in examined tissues in both cultivated and wild subspecies. Transcriptomic analysis of three genotypes showed that most of *MeHDZ* genes responded differently to drought and polyethylene glycol treatments. Subsequently, quantitative RT-PCR analysis revealed comprehensive responses of twelve selected *MeHDZ* genes to various stimuli including cold, salt, and ABA treatments. These findings will increase our understanding of *HD-Zip* gene family involved in abiotic stresses and signaling transduction, and will provide a solid base for further functional characterization of *MeHDZ* genes in cassava.

## Introduction

Homeodomain-leucine zipper (HD-Zip) proteins are one of key transcription factors in plants. Based on sequence analysis, HD-Zip proteins were characterized by the presence of two functional domains: a homeodomain (HD) responsible for specific binding to DNA and a leucine zipper (LZ) motif which closely linked to HD and acted as a dimerization motif [1,2]. To date, many members of HD-Zip proteins have been found in a large number of plant species, including Arabidopsis [3], rice [4], maize [5], soybean [6], legume [7], banana [8], and so on. Based on the structures, HD-Zip proteins were mainly categorized into four groups: HD-Zip I to HD-Zip IV [2]. Members of HD-Zip I and II contained the conserved HD and LZ domains and bound to the similar sequence CAAT-N-ATTG [9]. In addition, HD-Zip II members contained other motifs, such as CPSCE [2]. Besides the HD and LZ domains, members of both HD-Zip III and IV also contained a START domain with putative lipid binding capability [10]. However, a conserved C-terminal MEKHLA motif, which was probably involved in oxygen redox and light signaling, was present in the members of HD-Zip III but absent in the HD-Zip IV subfamily [11].

HD-Zip proteins have been demonstrated to participate in many biological processes related to plant growth and development [1,2]. Members of HD-Zip I subfamily mainly participated in abiotic stresses such as drought, extreme temperatures, osmotic and light stresses [2,3]. For example, *AtHB5*, *-6*, *-7* and *-12*, which belonged to *Arabidopsis* HD-Zip I subfamily, were up-regulated or down-regulated by drought stress or externally applied abscisic acid (ABA) [12–15]. Other members of this subfamily, such as *AtHB52* and *AtHB53*, were up-regulated in dark condition [3]. HD-Zip II proteins were mainly involved in shade avoidance and auxin response [16]. For examples, *AtHB2* in this subfamily was specifically and reversibly regulated by red to far-red ratio, and it was participated in the regulation of *Arabidopsis* shade avoidance response [17]. *HAT2*, another member of HD-Zip II subfamily, was isolated as an auxin-inducible gene through DNA microarray screening [18]. HD-Zip III members mainly participated in the developmental regulation of apical meristem, vascular bundles, auxin transport and lateral organ initiation [2]. For examples, *AtHB8* and *AtHB15* were involved in vascular development [19,20]. Several studies have demonstrated that *REV*, *PHB* and *PHV*, along with *KANADI*, were involved in controlling the abaxial-adaxial patterning of lateral organs [21]. HD-Zip IV proteins generally played important roles in anthocyanin accumulation, epidermal cell differentiation, trichome formation and root development [1,2]. For examples, *atml1 pdf2* double mutants resulted in severe defects in shoot epidermal cell differentiation and failed to survive after germination [22]. *ANL2* affected anthocyanin accumulation in subepidermal tissues [23], while other members in this subfamily, such as *HDG11*, *HDG12* and *GL2*, were involved in trichome development [2,24]. Recently, many studies have demonstrated that HD-Zip III and IV subfamilies were also involved in abiotic stresses such as salt, drought and ABA treatment [6,8,25].

Cassava (*Manihot esculenta*) is one of the most important root crops in tropical and sub-tropical areas and provides staple food for over 700 million people around the world [26]. Due to its good performance under adverse conditions such as drought and low fertilization, cassava has been considered as an important food security crop worldwide. However, as a representative tropical crop, cassava is very sensitive to cold stress [27]. To date, the mechanisms underlying its abiotic stresses remain elusive. With the availability of the cassava genome sequences [28,29], it provides an excellent opportunity for genome-wide annotation, classification and comparative genome research. Although *HD-Zip* genes have been extensively characterized in many species, a systemic study of HD-Zip gene family (especially for the stress-responsive members) has not been performed in cassava. In this study, a total of 57 *HD-Zip* genes were identified and characterized in the cassava genome. Besides their basic characteristics including phylogenetic relationship, gene structure, conserved protein motif and genome location, the expression profiles of these *HD-Zip* genes were investigated in different organs of various genotypes and several stress conditions, including drought, polyethylene glycol (PEG), cold, salt, and ABA treatments through RNA-seq or quantitative RT-PCR (qRT-PCR). These findings will provide a useful foundation for further investigations into the molecular biological functions of the *HD-Zip* genes in cassava.

## Materials and methods

### Plant materials and stress treatments

To reveal the expression of *HD-Zip* genes either in different tissues or in response to drought stress in cassava, several previously generated RNA-seq data [30,31] were used. In total four cassava accessions, including one wild subspecies (W14, *M*. *esculenta* subsp. *flabellifolia*) and three cultivated varieties (Arg7, Ku50 and SC124), were used in this study. Of which, Arg7 is adapted to high-latitude geographical region of Argentina and of moderate drought-tolerance; SC124 is a widely planted cultivar in China and can survive in severe drought condition, while Ku50 is a representative cultivar with high root yield and high starch content in root tubers and can grow under unfavorable environmental conditions [29,32,33].

In cassava growing season, Arg7, Ku50 and W14 were planted in the farm of Chinese Academy of Tropical Agricultural Sciences (Haikou, China) in the summer of 2013. To detect the changes of gene expression in different cassava tissues, samples from leaf (90 days after planting), stem (90 days after planting), early storage root (ESR, 90 days after planting), middle storage root (MSR, 150 days after planting) and last storage root (LSR, 210 days after planting) were collected for RNA-seq analysis.

To investigate the changes of gene expression in response to drought stress, stems of about 15 cm in length and two to three buds were selected and planted vertically in pots (sand: vermiculite = 1: 1; height × upper diameter × bottom diameter = 18.8 × 18.5 × 14.8 cm), as previously described [34]. About 45 days later, two experiments were conducted: (1) seedlings of Arg7, SC124 and W14 were withheld water for 0 day (control) and 12 days, and their leaves and roots were collected for RNA-seq, respectively; (2) seedlings of Ku50 were watered by 20% polyethylene glycol (PEG) 6000 solution, then the leaves, including folded leaf (FL), full expanded leaf (FEL) and bottom leaf (BL), as well as root (RT) were collected at 0, 3, 24 h after the treatment for RNA-seq, respectively. Each sample was pooled from 15 plants.

To examine the response of cassava to different abiotic stresses, including cold, salt, and ABA treatment, 2-month-old cassava seedlings of Arg7 were used and their leaves were collected for qRT-PCR. For salt stress, cassava seedlings were watered with 300 mM NaCl solution for 24 days. For ABA treatment, cassava seedlings were sprayed with 100 μM ABA for 72 h. For cold stress, cassava seedlings were exposed to 4°C for 48 h, and then returned to normal growth conditions for 14 days of recovery.

### Identification of HD-Zip family genes in cassava

The whole genome and protein sequences of cassava were downloaded from phytozome database v9.0 (https://phytozome.jgi.doe.gov/). Hidden Markov Model (HMM) profiles of homeobox (HD, PF00046) and homeobox associated leucine zipper (HALZ, PF02183) were downloaded from PFam (http://pfam.sanger.ac.uk/) and were used as queries to identify putative cassava *HD-Zip* genes by local HMM-based searches setting E-values < 0.01 using HMMER3 [35]. Additionally, protein sequences of *HD-Zip* genes from *Arabidopsis* and rice [36] were used in a BLAST search using BLASTP against cassava proteins to explore more HD-Zips which might be missed by HMM profile searching. After removing redundant sequences, each HD-Zip candidate was further confirmed by investigating conserved domains using database of CDD (http://www.ncbi.nlm.nih.gov/Structure/bwrpsb/bwrpsb.cgi) in automatic mode (threshold = 0.01, maximum hits = 500) and PFam setting E-value equal to 1.0, respectively. The molecular weight and isoelectric points of each HD-Zip protein were calculated using ExPASy tools (http://www.expasy.org/tools/).

### Phylogenetic and sequence analysis

Besides HD-Zips from *Arabidopsis* and rice, protein sequences of published *HD-Zip* genes from *Vitis vinifera*, *Medicago truncatula*, *Populus trichocarpa*, and maize [5,36] were also used. Protein sequences of *HD-Zip* genes were aligned using MUSCLE [37]. Phylogenetic tree was conducted in MEGA6 [38] using Maximum-Likelihood method with Jones-Taylor-Thornton (JTT) amino acid substitution model. Bootstrap analysis was performed using 1000 replicates with the partial deletion model for gaps/missing data.

HD-Zip exon-intron structure was performed with Gene Structure Display Server (GSDS, http://gsds.cbi.pku.edu.cn/) [39] by aligning cDNA to their corresponding genomic DNA sequences. Protein motifs were predicted by MEME software [40] with optimum motif width ranged from 11 to 50 and setting the maximum number of motifs equal to 15. The predicted motifs of HD-Zip proteins were annotated by searching against InterProScan database (http://www.ebi.ac.uk/Tools/pfa/iprscan/).

To demonstrate the possible regulatory mechanism of *HD-Zip* genes in various stress responses, 1500 bp promoter region upstream of the transcription start site (ATG) was used to analyze cis-elements using PlantCARE database (http://bioinformatics.psb.ugent.be/webtools/plantcare/html/). The overrepresentation analysis of cis-elements was performed by exact binominal test in each subfamily of *HD-Zip* genes.

### Chromosomal location and gene duplication

Cassava *HD-Zip* (referred to *MeHDZ*) genes were mapped on cassava chromosomes according to their positions in the early genome version 6.1 from phytozome database (https://phytozome.jgi.doe.gov/). The schematic diagram of *MeHDZ* genes was drawn by MapInspect (http://www.plantbreeding.wur.nl/UK/software_mapinspect.html).

Tandem duplicates were defined as genes that occurred within a 50 kb region. Segmental duplicates were detected by BLASTN (score < 1e-5) using 100 kb (containing 50 kb upstream and 50 kb downstream) sequences flanking to the genes, and were further confirmed by chaining alignments (alignment length > 200 bp and sequence identity > 85%) [41].

### Transcriptome and qRT-PCR analysis

Total RNA was extracted using RNA plant reagent kits (Tiangen Company, Beijing, China). Each RNA-Seq library was constructed as previously described in Yang et al [42]. Then, libraries were indexed, pooled and sequenced on Illumina HiSeq2000. Transcriptome analysis was performed as previously described [33,34]. For examples, sequence quality was examined using FastQC (http://www.bioinformatics.babraham.ac.uk/projects/fastqc/), clean reads were mapped to cassava genome (version 4.1) using Tophat v2.0.13 [43], and raw count data were obtained and normalized by Cuffdiff embedded in Cufflinks pipeline v2.1.1 [44]. The transcriptome data were submitted to NCBI database and their accessions were shown in S1 Table.

Expression of *MeHDZ* genes in response to various stimuli, including cold, salt, and ABA treatments, was examined by qRT-PCR method using SYBR-green (TaKaRa Biotechnology Co. Ltd, Dalian, China) and Stratagene Mx3005P system (Stratagene, CA, USA) as previously described [34]. The cassava actin gene [45] was used as an internal control. The specific primers of target *MeHDZ* genes were designed by Primer 5.0 software (S2 Table). Subsequently, the specificity of each primer pair was examined by qRT-PCR melting curve analysis, agarose gel electrophoresis, and PCR products sequencing [30]. For each sample, qRT-PCR reaction was performed with three independent biological replicates and the relative mRNA expression level was calculated by 2^-ΔΔCt^ as before [42]. Statistical difference was examined by Duncan’s multiple range test (n = 3) and expression values with different letters were significant at *P* < 0.05.

## Results

### Identification of *HD-Zip* genes in cassava

In total, 125 HD-Zip candidate sequences were found through searching against cassava genome using HMM and BLASTP methods. After removing redundant sequences and confirming the presence of both HD and LZ domains by CDD and PFam databases, 57 non-redundant *HD-Zip* genes (designated as *MeHDZ01-57*) were finally retained and used for further analysis. These proteins varied from 79 (MeHDZ57) to 853 (MeHDZ01) amino acid in length, and their molecular weight ranged from 9.12 (MeHDZ05) to 93.83 (MeHDZ13) kDa (S3 Table).

### Phylogenetic and evolutionary analysis of *HD-Zip* genes

**Fig 1:**
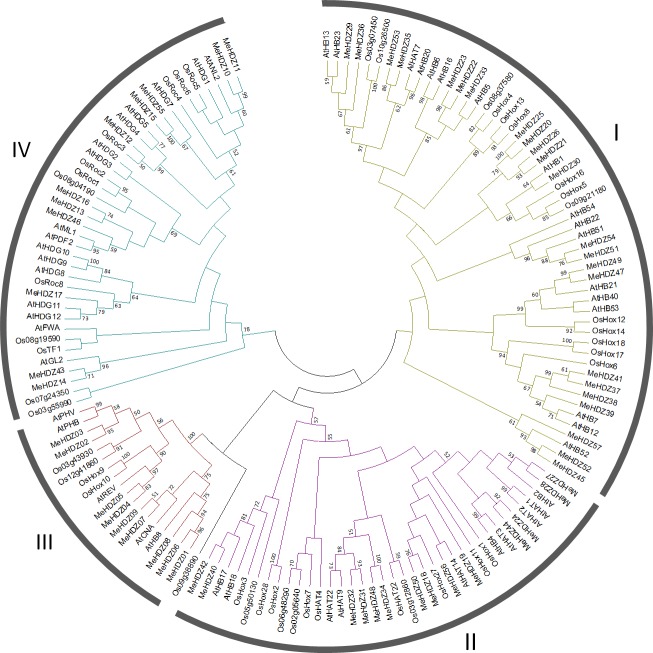
Phylogenetic clustering of HD-Zip proteins from Arabidopsis, rice and cassava. The unrooted phylogenetic tree was constructed by Maximum-Likelihood method with 1000 bootstrap replicates. The HD-Zip protein sequences corresponding to 44 from rice (prefixed with ‘Os’), 48 from *Arabidopsis* (prefixed with ‘At’), and 57 from cassava (prefixed with ‘Me’) were well separated into four major groups (I-IV).

To study the evolutionary relationship of *HD-Zip* genes between cassava and other species, protein sequences of 57 *MeHDZ* genes, together with 48 from *Arabidopsis* and 44 from rice [36], were aligned and used for phylogenetic analysis (S4 Table and S1 File). As shown in Fig 1, phylogenetic tree well clustered these MeHDZ proteins into four groups (subfamily I-IV), together with known HD-Zip I to IV members of rice and *Arabidopsis* respectively in each clade, indicating that there were four major types of *MeHDZ* genes in cassava.

In addition, HD-Zip proteins from seven species including five eudicots (*Arabidopsis*, cassava, *Vitis vinifera*, *Medicago truncatula*, and *Populus trichocarpa*) and two monocots (rice and maize) were also aligned and further divided into subclasses (S1 Fig and S2 File), based on which four different evolutionary scenarios were revealed: (1) *HD-Zip* genes that were generated before the divergence of eudicots and monocots and they were highly conserved, as they were presented in all of the seven species although the number of *HD-Zip* genes might be varied. Moreover, this phenomenon was observed in each subfamily, e.g., subclass *a*, *f*, *i* in subfamily I, subclass *m*, *p* in subfamily II, subclass *t* in subfamily III, and subclass *D* in subfamily IV; (2) *HD-Zip* genes that were generated before the divergence of eudicots and monocots but their homologs in some speices might be lost during the evolution, because they were presented in both eudicots and monocots but not in all of examined species, e.g., subclass *j*, *k* in subfamily II, subclass *q*, *r* in subfamily III, and subclass *u*, *w* in subfamily IV; (3) *HD-Zip* genes that were generated after the divergence of eudicots and monocots, as they were exclusively presented in eudicots. They were either highly conserved (e.g, subclass *c*, *h*, *n*, *z*, *A*, and *C* containing *HD-Zip* genes presented in all of five examined eudicots) or not very conserved (e.g, subclass *b*, *d*, *s*, and *y* containing *HD-Zip* genes only presented in partial of examined eudicots); (4) *HD-Zip* genes that were generated after the divergence of eudicots and monocots, but they were exclusively presented in monocots. For examples, subclass *g*, *l*, *o*, *x*, *B*, and *F* only contained *HD-Zip* genes from rice and maize in this study. Taken together, these results indicated a complicated evolutionary process of *HD-Zip* genes in plants.

### Gene structure and conserved motif analysis

**Fig 2:**
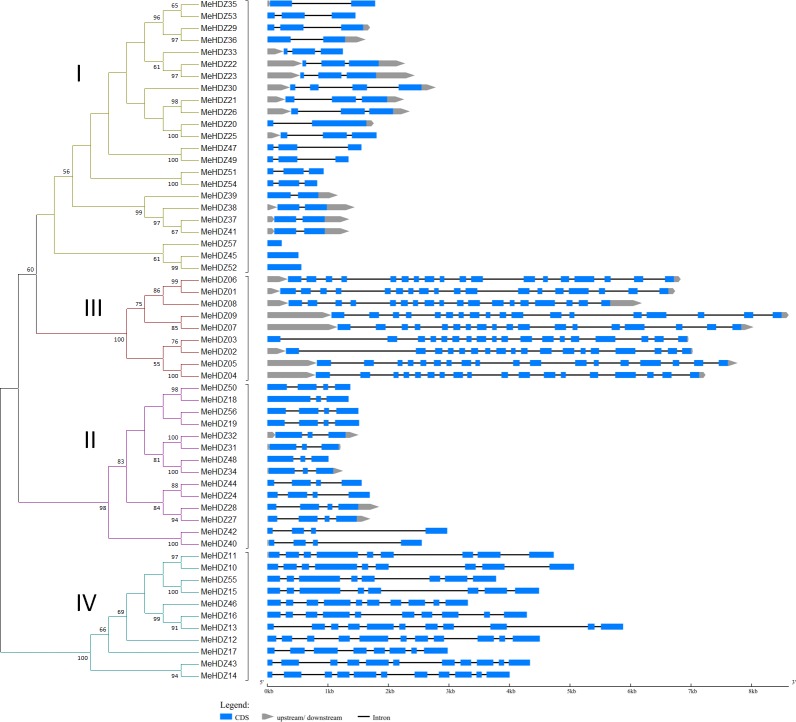
Exon-intron structure of 57 *MeHDZ* genes in cassava. The phylogenetic tree was constructed by Maximum-Likelihood method with 1000 bootstrap replicates. The *MeHDZ* genes were clustered into four groups, I to IV. Exon-intron analysis was performed using GSDS (http://gsds.cbi.pku.edu.cn/). Length of exons and introns of each *MeHDZ* gene was displayed proportionally. Upstream was defined as the sequences before the transcription start site ‘ATG’, while downstream was defined as the sequences after the stop codon (e.g., ‘TAA’).

To gain information regarding the gene structure of *MeHDZ* genes, exon-intron structure analysis was performed for each *MeHDZ* gene individually (Fig 2). Overall, most closely related members within the same subfamily shared similar exon-intron structure and intron numbers. There were 23 members clustered in MeHDZ I subfamily and most of them contained one to three exons. There were 14 members in MeHDZ II subfamily, and they contained three to four exons. There were 9 and 11 members in MeHDZ III and IV subfamilies, respectively. Compared with MeHDZ I and II subfamilies, much more exons were found in MeHDZ III (18 in each) and IV (9.6 on average) subfamilies, respectively.

**Fig 3:**
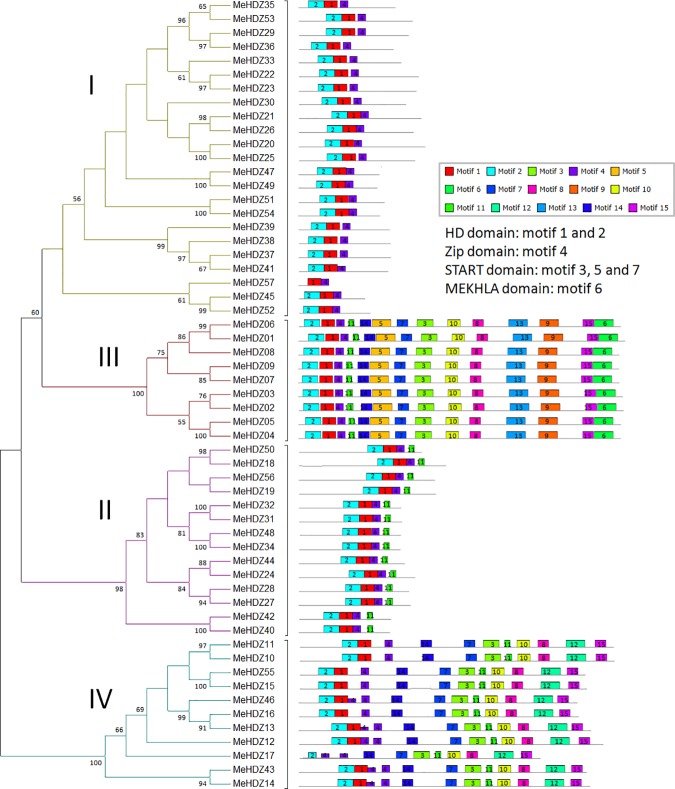
Conserved motifs of MeHDZ proteins corresponding to their phylogenetic relationships. The conserved motifs were identified by MEME software. Motifs were indicated by different colored boxes with the motif number, while non-conserved sequences were represented by grey lines. Length of motifs was exhibited proportionally.

In total, 15 conserved motifs were captured by MEME software and subsequently annotated with InterProScan database (Fig 3, S2 Fig and S5 Table). As expected, HD domain (motif 1 and 2) and leucine zipper domain (motif 4) were found in all identified MeHDZ members. Some motifs that presented only in certain MeHDZ subfamilies were observed. For examples, START domain, which was represented by motif 3, 5 and 7, was not found in members of MeHDZ I and II subfamilies but presented in all members of MeHDZ III and IV subfamilies; MEKHLA domain, represented by motif 6, was presented only in members of MeHDZ III subfamily. Besides the well annotated, some unknown motifs (e.g., motif 9) were also found specifically in some subfamilies, indicating that these motifs might be key elements for the subfamily-specific functions.

### Chromosomal location and gene duplication of *MeHDZ* genes

**Fig 4:**
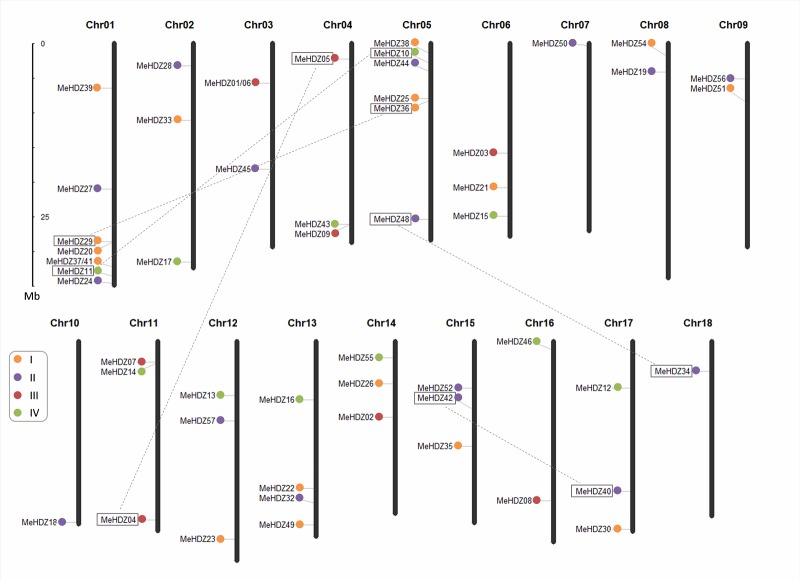
Physical locations of *MeHDZ* genes on cassava chromosomes. Chromosome numbers were indicated at the top of each chromosome. *MeHDZ* genes from different subfamilies were indicated by different colors. Five pairs of *MeHDZ* genes connected by dash lines were resulted from segmental duplication.

Genomic distribution of *MeHDZ* genes was determined by their chromosomal positions. To the best of our knowledge, this is the first report concerning the genomic distribution of gene family in cassava. Except for three members of *MeHDZ31*, *-47* and *-53* that were located on scaffolds, the remaining 54 *MeHDZ* genes were mapped on 18 cassava chromosomes (Fig 4; S6 Table). *MeHDZ01* and *MeHDZ06*, together with *MeHDZ37* and *MeHDZ41*, which may result from different alternative splicing, were mapped to the same loci on chromosome 3 and 1, respectively. Overall, an unequal distribution of *MeHDZ* genes was revealed in cassava. For example, a maximum of eight *MeHDZ* genes were presented on chromosome 1, in contrast, only one *MeHDZ* gene was respectively located on chromosome 7, 10 and 18, which contained the minimum *MeHDZ* gene in cassava (Fig 4).

Segmental duplication and tandem duplication, which played important roles in expanding new members during the evolution of a gene family, were investigated to elucidate the potential evolution mechanism of *MeHDZ* genes in cassava. Totally, ten *MeHDZ* genes, forming five pairs, were identified as segmental duplications (Fig 4; S3 Fig). Of which, two pairs were from MeHDZ II subfamily, while the other three pairs were found in the remaining three subfamilies, respectively. However, none of the *MeHDZ* genes seemed to be generated from tandem duplications in our analysis. These results implicated that segmental duplication events were the main cause for the expansion of *MeHDZ* genes in cassava.

### Cis-element identification of *MeHDZ* genes

To demonstrate the possible regulatory mechanism of *MeHDZ* genes, 1.5 kb promoter sequences upstream of the transcription start site (ATG) were extracted and used for cis-element prediction. As expected, many stress-related elements, such as MBS for drought, HSE for heat, LTR for low-temperature, and WUN-motif for wound-response, were identified in the promoter region of *MeHDZ* genes (S7 Table). Additionally, many hormone-related cis-elements, including ABRE for ABA, TGA-element for auxin, ERE for ethylene, P-box and GARE-motif for gibberellic acid (GA), CGTCA-motif and TGACG-motif for methyl jasmonate (MeJA), and TCA-element for salicylic acid, were also found (S7 Table). Moreover, other cis-elements such as *circadian* for circadian control, ARE for anaerobic induction, and as much as 20 cis-elements for light response were also observed.

To explore the particular roles of *MeHDZ* genes in each subfamily, overrepresentation analysis was conducted respectively for these cis-elements, and subfamily-specific overrepresented manners were revealed (S7 Table). Several cis-elements related to abiotic stress, e.g., ABRE, HSE, and TC-rich repeats, were significantly overrepresented in MeHDZ I and II subfamilies, while GARE-motif and MBS were overrepresented in MeHDZ III and IV subfamilies, respectively. Further, light responsive elements (e.g., Sp1 and G-box) also exhibited subfamily-specific overrepresented manners. Together, these results supported that subfamily-specific roles of HD-Zip subfamilies might be related to the regulatory elements in the promoter region.

### Expression profiles of *MeHDZ* genes in different tissues of three genotypes

**Fig 5:**
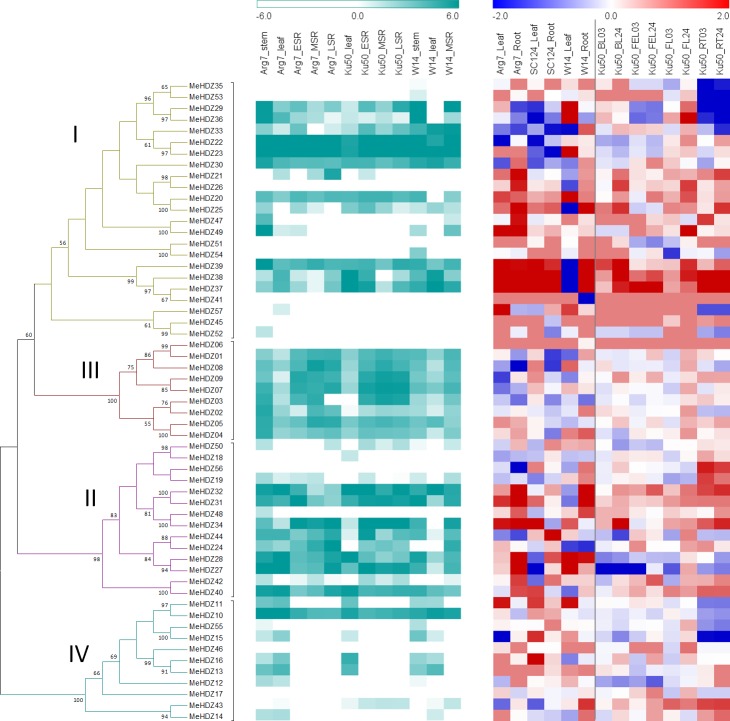
Expression profiles of *MeHDZ* genes in cassava. (A) Expression of *MeHDZ* genes in different tissues of three genotypes. Log2-transformed FPKM value was used to plot the heatmap. ESR: early storage root; MSR: middle storage root; LSR: last storage root. (B) Expression of *MeHDZ* genes in response to drought and PEG treatments. Log2 based fold change of treatment/control was used to plot the heatmap. FL: folded leaf; FEL: full expanded leaf; BL: bottom leaf; RT: root. The numbers attached behind samples represented the time points at which samples were collected: e.g., 03 and 24 represented 3 and 24 h, respectively.

To reveal expression profiles of *HD-Zip* genes in different tissues, previous generated RNA-seq for the samples of stem, leaf, early storage root (ESR), middle storage root (MSR), and last storage root (LSR) in one wild subspecies (W14) and two cultivars (Arg7 and Ku50) was used. Expressed genes were arbitrarily defined as those with FPKM (Fragments Per Kilobase of exon per Million fragments mapped) > 1. Overall, different expression profiles were revealed within each subfamily of *MeHDZ* genes (Fig 5A; S8 Table). In MeHDZ I subfamily, most genes were expressed in all tested organs, of which *MeHDZ22* and *MeHDZ23* showed the highest expression level. Genes such as *MeHDZ21*, *-47*, *-49* and *-54* were expressed only in a few samples, while the others such as *MeHDZ26*, *-41* and *-51* were not expressed at all. Similar gene expression patterns were observed in MeHDZ II subfamily. For example, *MeHDZ27*, *-28*, *-31*, *-32*, *-40* and *-44* were expressed in all tested samples, while *MeHDZ56* were not expressed and the remaining genes were expressed in partial samples. Compared with MeHDZ I and II subfamilies, most genes in MeHDZ III and IV subfamilies exhibited distinct tissue-specific expression patterns. For examples, eight out of nine genes (except *MeHDZ06* that was not expressed in any samples) from MeHDZ III subfamily were lower expressed in leaves than in other tissues; and seven out of eleven genes (except *MeHDZ10* that showed constitutive expression, *MeHDZ17* and *MeHDZ46* that were not expressed, and *MeHDZ43* that expressed higher in root than in other samples) from MeHDZ IV subfamily were lower expressed in roots than in others (Fig 5A). Taken together, these results suggested that the functions of *MeHDZ* genes were diverged through constitutive expression, non-expression, or tissue-specific expression during cassava evolution processes.

Different expression patterns were also revealed in the same tissue among different genotypes, especially between the cultivated and the wild subspecies. Compared to W14, the expression of 22, 25 and 15 *HD-Zip* genes was greatly changed (absolute value of fold-change > 2) in stem, leaf and MSR in Arg7, while 15 and 17 *HD-Zip* genes were differentially expressed in leaf and MSR in Ku50, respectively (S8 Table).

### Expression analysis of *MeHDZ* genes responding to drought in different genotypes

To demonstrate the responses of *MeHDZ* genes to drought stress, seedlings of one wild subspecies (W14) and two cultivated varieties (Arg7 and SC124) were subjected to 12-day water withholding treatment, and their leaf and root samples of control and drought-treated plants were collected for RNA-seq analysis, respectively. As shown in Fig 5B, the expression of 18 and 24 genes were greatly changed (absolute value of fold-change > 2) in leaf and root in Arg7, respectively. Comparable number of genes were observed in leaf in SC124 (19) and W14 (21), however, the number was decreased to about one-half in root, e.g., 13 genes in SC124 and 14 genes in W14, respectively (S9 Table). These results suggested that more *MeHDZ* genes were triggered by drought stress in the root of SC124 and W14 than that in Arg7, which might be one explanation for the good performance of SC124 and W14 relative to Arg7 under drought condition. It’s worthy to note that *MeHDZ* genes showed different response to drought between wild subspecies and cultivated varieties. For example, in leaf, *MeHDZ25*, *-39*, *-38* and *-37* from MeHDZ I subfamily were greatly depressed in W14 but extremely induced in Arg7 and SC124. On the contrary, the expression of *MeHDZ36* and *MeHDZ23* was greatly increased in leaf of W14 but decreased in that of SC124 (Fig 5B). The results implied that *MeHDZ* genes might play different roles in drought stress between wild subspecies and cultivated varieties. As expected, the expression of most members of MeHDZ IV subfamily was not changed in root, which was consistent with their root-specific non-expressed patterns (Fig 5A).

In addition, PEG solution, which is usually used to simulate drought stress, was applied to Ku50 seedlings to investigate the expression profiles of *MeHDZ* genes (S10 Table). Overall, the expression of most genes was more dramatically changed at 24 h than that at 3 h (Fig 5B), suggesting that longer periods of drought stress caused more serious influences on cassava. Consistent with water withholding treatment, genes such as *MeHDZ20*, *-25*, *-21*, *-26*, *-39*, *-38*, and *-37* from MeHDZ I subfamily, and *MeHDZ31*, *-32* and *-34* from MeHDZ II subfamily, exhibited strong induction in the majority of tested samples. On the contrary, *MeHDZ53*, *-35*, *-29* and *-36* from MeHDZ I subfamily, and *MeHDZ10*, *-11*, *-15* and *-55* from MeHDZ IV subfamily were greatly depressed in root (Fig 5B). Taken together, these results indicated that all four MeHDZ subfamilies were responsive to drought stress and the expression analysis will provide useful clues for further characterizing their functions in cassava.

### Expression profiles of *MeHDZ* genes in response to various treatments

Besides HD-Zip I members that were well demonstrated to associate with abiotic stress, it was of great interests to investigate whether genes from HD-Zip II to IV were involved in abiotic stress responses as previously proposed. Here twelve genes (including three from each MeHDZ subfamily), which exhibited similar expression patterns among different tissues within subfamily and differentially expressed under drought or PEG treatments based on RNA-seq data, were chosen to further examine their responses to cold, salt, and ABA treatment through qRT-PCR method.

**Fig 6:**
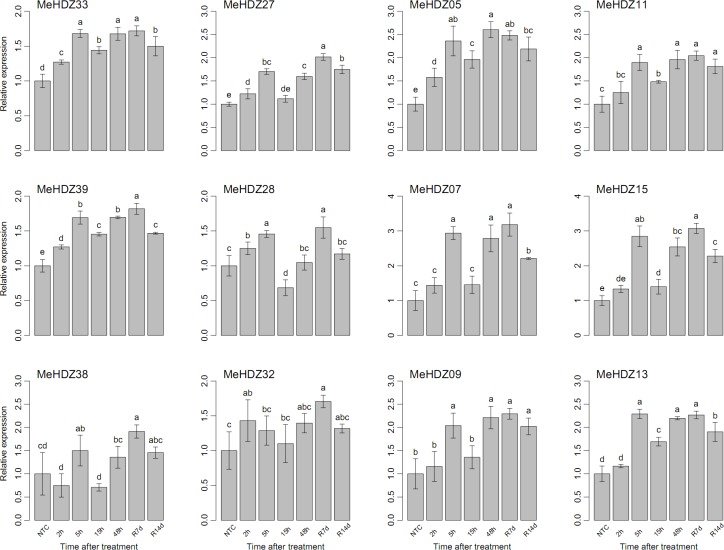
Relative expression of *MeHDZ* genes in leaves under cold treatment. NTC (no treatment control) was normalized as “1” at each graph. Data were shown as mean ± standard deviation derived from three biological replicates, and values with different letters were significant based on Duncan’s multiple range tests (*P* < 0.05, n = 3).

Under cold treatment following recovery, the majority of tested *MeHDZ* genes showed similar expression patterns, and they were significantly induced at 5h and depressed at 15h, and then increased from 48h to R7d and finally decreased at R14d (Fig 6).

**Fig 7:**
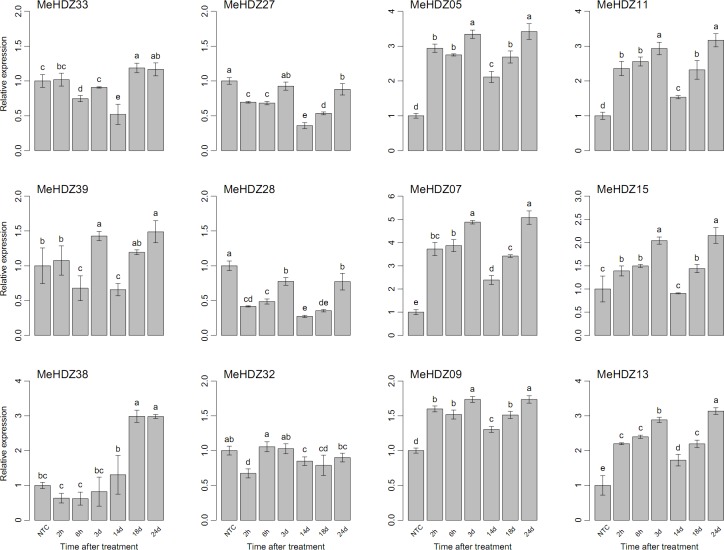
Relative expression of *MeHDZ* genes in leaves under NaCl treatment. NTC (no treatment control) was normalized as “1” at each graph. Data were shown as mean ± standard deviation derived from three biological replicates, and values with different letters were significant based on Duncan’s multiple range tests (*P* < 0.05, n = 3).

Under salt treatment, three members of MeHDZ I subfamily exhibited different expression trends, e.g., *MeHDZ33* was significantly decreased from 6h to 14d but increased at 18d and 24d, *MeHDZ39* was greatly induced at 3d and 24d, while *MeHDZ38* was almost not changed before 14d but dramatically increased at 18d and 24d (Fig 7). *MeHDZ27* and *MeHDZ28* from MeHDZ II subfamily were significantly depressed, especially at 2-6h and 14-18d. Interestingly, members from MeHDZ III and IV subfamilies exhibited similar expression patterns and their expression was greatly induced from 2h to 3d, followed by a significant decrease at 14d and then increased at 18d and 24d (Fig 7).

**Fig 8:**
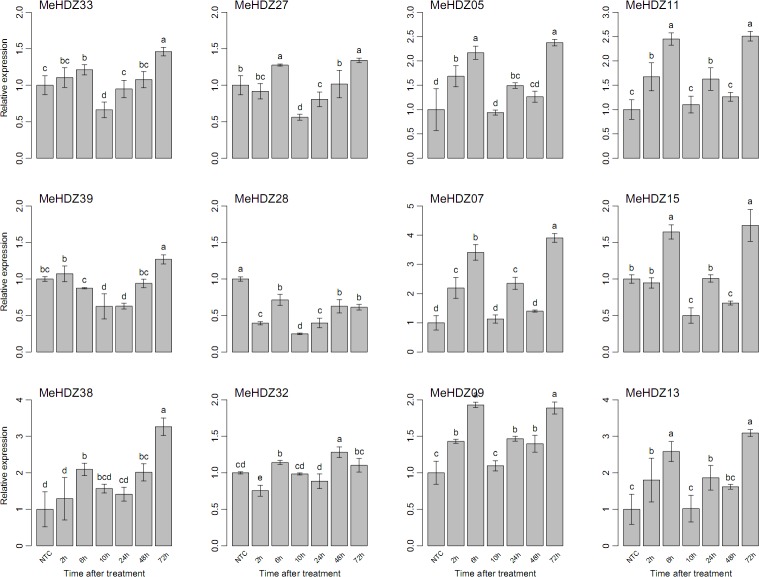
Relative expression of *MeHDZ* genes in leaves under ABA treatment. NTC (no treatment control) was normalized as “1” at each graph. Data were shown as mean ± standard deviation derived from three biological replicates, and values with different letters were significant based on Duncan’s multiple range tests (*P* < 0.05, n = 3).

Under ABA treatment, the expression of *MeHDZ33* and *MeHDZ39* from MeHDZ I subfamily and *MeHDZ27* from MeHDZ II subfamily was greatly depressed at 10h but induced at 72h. The expression of *MeHDZ38* from MeHDZ I subfamily was not dramatically induced until at 72h, while the expression of *MeHDZ28* from MeHDZ II subfamily was greatly depressed during the whole treatment (Fig 8). Interestingly, members from MeHDZ III and IV subfamilies showed very similar expression trends and their expression was greatly induced from 2h to 6h, followed by a significant decrease from 10h to 48h and then dramatically increased at 72h (Fig 8).

Together, these results indicated that similar to HD-Zip I, members from HD-Zip II and IV subfamilies were also involved in abiotic stresses (e.g., cold and salt) and ABA response in cassava.

## Discussion

Cassava is an important crop for tropical and sub-tropical regions. It has good performance even grown under unfavorable environments (e.g., drought), however, the mechanisms underlying its abiotic stresses tolerance remain elusive. The ABA signaling pathway has well been demonstrated to play crucial roles in plant abiotic stresses, in which HD-Zips are important transcription factors involved in ABA signaling pathway. Many researches have further confirmed the involvement of HD-Zip I genes in abiotic stresses (e.g., drought, cold and salt) and ABA signaling response [2,3]. However, the identity, characterization and function of *HD-Zip* genes are still unknown in cassava.

In this study, a comprehensive analysis of *HD-Zip* genes was performed in cassava genome, and a total of 57 *HD-Zip* genes were identified. With the availability of complete genome sequences, it enabled detailed comparisons of *HD-Zip* genes among many species. Compared with cassava, comparable number of *HD-Zip* genes was found in maize (55) [5], although this number was higher in soybean (88) [6] but less in peach (33) [46], rice (44) [36] and *Arabidopsis* (48) [36]. Similar to *Arabidopsis* and rice, HD-Zip proteins were well classified into four subfamilies in cassava (Fig 1). Besides, the same number of HD-Zip subfamilies was also found in other monocot and dicot plants (e.g., maize, soybean and peach) [5,6,46]. This classification was well supported by gene structure analysis. For example, in cassava, the members of MeHDZ III subfamily had the highest numbers of exons (18), then followed by MeHDZ IV (8 to 11), I (1 to 4) and II (3 to 4) subfamilies (Fig 2). These findings were similar to that reported in maize, soybean and peach [5,6,46]. Conserved motif comparisons also supported the classification of *HD-Zip* genes. For example, in cassava, members of MeHDZ I and II subfamilies had less conserved motifs comparing to the members of MeHDZ III and IV subfamilies. Moreover, distinct motifs (e.g., START and MEKHLA domain) were exclusively found in the members of MeHDZ III and IV but not in MeHDZ I and II subfamilies (Fig 3). Similar conclusions were observed in many monocot and dicot plants such as *Arabidopsis*, rice, maize, soybean and peach [3–6,46]. Together, *MeHDZ* genes of the same subfamilies shared similar characteristics of gene structure and conserved motif, supporting the classification of MeHDZ subfamilies.

Gene duplication, through either segmental duplication or tandem duplication, played important roles in expansion of new members during the evolution of a gene family [47]. Duplicated genes usually have high levels of sequence similarities, however, over the course of evolution, the fates of duplicated genes are quite different: either lose its function (nonfunctionalization), or obtain new function (neofunctionalization), or keep the ancestral function (subfunctionalization) [48]. Comparison of expression profiles of cassava *MeHDZ* genes involved in tandem and segmental duplications would reveal similar, different or silenced gene expression relative to other members, signifying conservation, neofunctionalization and nonfunctionalization after the duplication events [48]. As expected, diverse expression patterns were revealed in different pairs of duplicated *MeHDZ* genes. For example, in MeHDZ I subfamily, *MeHDZ29* and *MeHDZ36* were duplicated genes and exhibited similar expression behavior, suggesting their conservation in the regulation of gene expression; in MeHDZ IV subfamily, *MeHDZ10* and *MeHDZ11* showed different expression patterns, indicating their divergence in gene expression and they acquired novel regulatory characteristic (e.g., function in different tissues); in MeHDZ II subfamily, *MeHDZ34* exhibited a constitutive expression while *MeHDZ48* was not expressed in almost all examined tissues (except for stem in Arg7) (Fig 5A), leading to nonfunctionalization after the duplication events. Similar results were also observed in *HD-Zip* genes from other plants [3,6].

Cis-elements played crucial roles in various aspects of biological processes through regulating gene expression. Several cis-elements related to abiotic stress (e.g., drought, low temperature and light) and response of hormones (e.g., ABA and auxin) were revealed in the promoter region of *MeHDZ* genes. This was coincident with the well characterized functions of *HD-Zip* genes in plants [2]. For example, members of HD-Zip I have been demonstrated to be involved in abiotic stresses such as drought/osmotic stresses [2,3]. Consistently, the expression of many members from MeHDZ I subfamily was significantly changed (either up-regulated or down-regulated) in three different genotypes under drought and PEG treatments in our study (Fig 5). Compared with HD-Zip I, the remaining three groups (II to IV) of *HD-Zip* genes were well characterized as they were involved in shade avoidance, auxin response, development and cell differentiation, rather than drought response, although a few evidences have showed that the expression of HD-Zip II to IV genes was up-regulated in response to water deficit [8,49,50]. In this study, the expression of many members from the MeHDZ II to IV subfamilies was dramatically increased or decreased under drought and PEG treatments, and additionally drought-inducible cis-element (e.g., MBS) was identified in these members, supporting possible roles of genes from MeHDZ II to IV subfamilies in response to drought stress in cassava.

Besides drought, lots of evidences have also revealed the involvement of HD-Zip I genes in other abiotic stresses (e.g., cold and salt) and related signal transduction pathways (e.g., ABA) [3]. However, no such information was available for the *MeHDZ* genes in response to various stimuli in cassava. Moreover, as previously proposed [51], it was of great interests to examine whether genes from HD-Zip II to IV were involved in abiotic stress responses. Thus, three genes of each MeHDZ subfamily were selected to further examine their expression in response to various treatments (Figs 6–8).

Two genes of MeHDZ I subfamily, *MeHDZ38* and *MeHDZ39* that were homologues of *AtHB12* and *AtHB7* respectively, were significantly induced under all tested treatments (Figs 6–8). This was consistent with previous reports that the expression of both *AtHB12* and *AtHB7* was greatly up-regulated in ABA, salt and cold treatments [3]. Similar results were also observed in *Medicago truncatula* where the homologues of *AtHB7* and *AtHB12* were induced under salt stress [52]. However, for another MeHDZ I subfamily gene, *MeHDZ33*, whose expression was also up-regulated in tested treatments, the expression of its *Arabidopsis* homologue (*AtHB16*) was decreased in salt and cold but not responded to ABA [3].

Overall, three examined genes of MeHDZ II subfamily were greatly induced by cold and depressed by salt stress, however, diverse responses were revealed under ABA treatment (Figs 6–8). In soybean, 14 and 16 out of 27 HD-Zip II genes significantly responded to salt and drought stresses, respectively [6]. Likely, HD-Zip II members of *Craterostigma plantagineum* were shown to be regulated by ABA and water availability [53]. In addition, the transcripts of HD-Zip II members from *Arabidopsis* and rice were dramatically changed upon drought exposure [4,49]. Together, these results suggested that HD-Zip II genes would play important roles in abiotic stresses.

Uniformly, each three tested members of MeHDZ III and IV subfamilies were all significantly induced in response to cold, salt and ABA treatments. Similar results were revealed in soybean that each six out of eight differentially expressed HD-Zip III genes were up-regulated by salt and drought, while eleven and nine out of eleven differentially expressed HD-Zip IV genes were up-regulated by salt and drought, respectively [6]. A recent study also revealed that three members (*REV*, *PHB* and *PHV*) of HD-Zip III subfamily in *Arabidopsis* were strongly decreased after three hours of ABA treatment [25]. These results indicated the involvement of HD-Zip III and IV genes in abiotic stresses and ABA response.

In conclusion, a total of 57 *HD-Zip* genes from cassava genome were identified, and their basic classification, gene structure, conserved protein motifs and evolutionary characteristics were revealed. Subsequently, the cis-elements in the promoter region were explored, and the expression profiles were investigated in different organs of three cassava genotypes, as well as in response to various stimuli through transcriptome or qRT-PCR analysis. These findings will provide a solid base for future studies on the functional characterization of *HD-Zip* genes, and will improve our understanding of *MeHDZ* genes involved abiotic stress and signaling transduction in cassava.

## Supporting information

S1 Fig Phylogenetic relationships of HD-Zip proteins from *Arabidopsis*, cassava, *Vitis vinifera*, *Medicago truncatula*, *Populus trichocarpa*, rice, and maize. Each HD-Zip subfamily was further divided into subclasses (from *a* to *F*). The HD-Zip protein sequences were prefixed with ‘At’ for *Arabidopsis*, ‘Me’ for cassava, ‘Vv’ for *Vitis vinifera*, ‘Mt’ for *Medicago truncatula*, ‘Pt’ for *Populus trichocarpa*, ‘Os’ for rice, and ‘Zm’ for maize, respectively.
(TIF)

S2 Fig Logos of 15 conserved amino acid motifs identified in the MeHDZ proteins. (TIF)

S3 Fig Pairwise chaining alignment results of *MeHDZ* genes in cassava. Red dots represented the relative positions of *MeHDZ* genes indicated at the bottom. Each 50 kb sequences upstream and downstream of the genes were selected to BLASTN against each other, and alignments with length > 200 bp and sequence identity > 85% were chained. Five pairs of *MeHDZ* genes identified as segmental duplications were marked with red boxes.
(TIF)

S1 File Protein sequence alignment of *HD-Zip* genes from *Arabidopsis*, rice, and cassava. (TXT)

S2 File Protein sequence alignment of *HD-Zip* genes from *Arabidopsis*, cassava, *Vitis vinifera*, *Medicago truncatula*, *Populus trichocarpa*, rice, and maize. (TXT)

S1 Table The accession numbers of transcriptomic data in NCBI. (XLSX)

S2 Table The specific primers of target *MeHDZ* genes used for qRT-PCR. (XLSX)

S3 Table General information of *MeHDZ* genes identified in this study. (XLSX)

S4 Table *HD-Zip* genes of each subfamily in cassava, *Arabidopsis* and rice. (XLSX)

S5 Table Conserved amino acid motifs and the annotations in *MeHDZ* genes. (XLSX)

S6 Table Chromosome locations of *MeHDZ* genes in cassava. (XLSX)

S7 Table Identification of cis-elements in the promoter region of *MeHDZ* genes. (XLSX)

S8 Table Expression of *MeHDZ* genes in different tissues of three cassava genotypes. (XLSX)

S9 Table Log2 based fold-change (treatment/control) of expression in leaf and root under drought treatment. (XLSX)

S10 Table Log2 based fold-change (treatment/control) of expression in leaf and root under PEG treatment. (XLSX)
