# Supplementary figures and images for: Genome-wide characterization and expression profiling of HD-Zip gene family related to abiotic stress in cassava
Source: PLoS One. 2017 Mar 1;12(3):e0173043. doi: 10.1371/journal.pone.0173043 (PMC5332091; doi:10.1371/journal.pone.0173043)

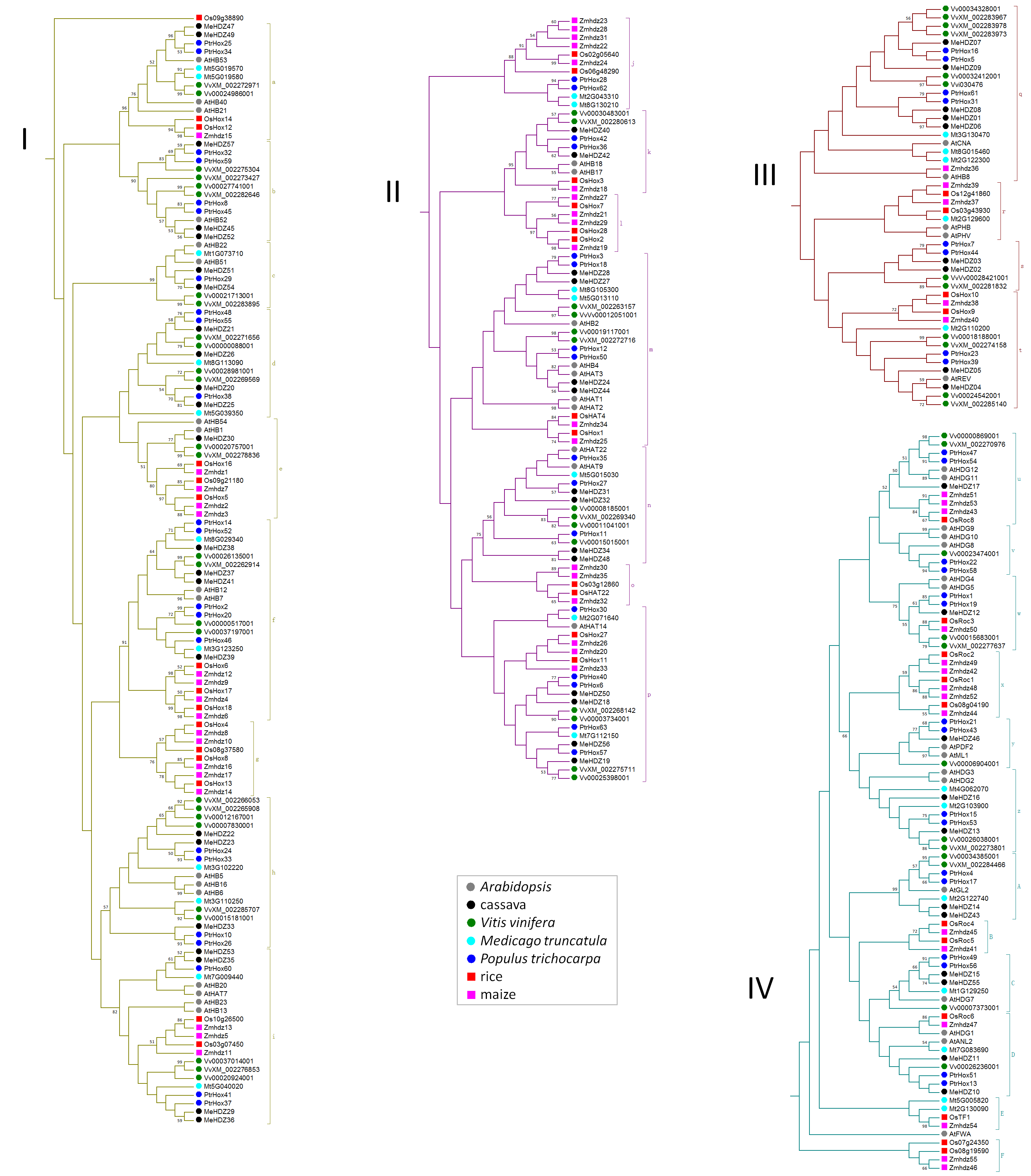

Supplement: S1 Fig — Each HD-Zip subfamily was further divided into subclasses (from a to F). The HD-Zip protein sequences were prefixed with ‘At’ for Arabidopsis, ‘Me’ for cassava, ‘Vv’ for Vitis vinifera, ‘Mt’ for Medicago truncatula, ‘Pt’ for Populus trichocarpa, ‘Os’ for rice, and ‘Zm’ for maize, respectively. (TIF) [file pone.0173043.s001.tif]

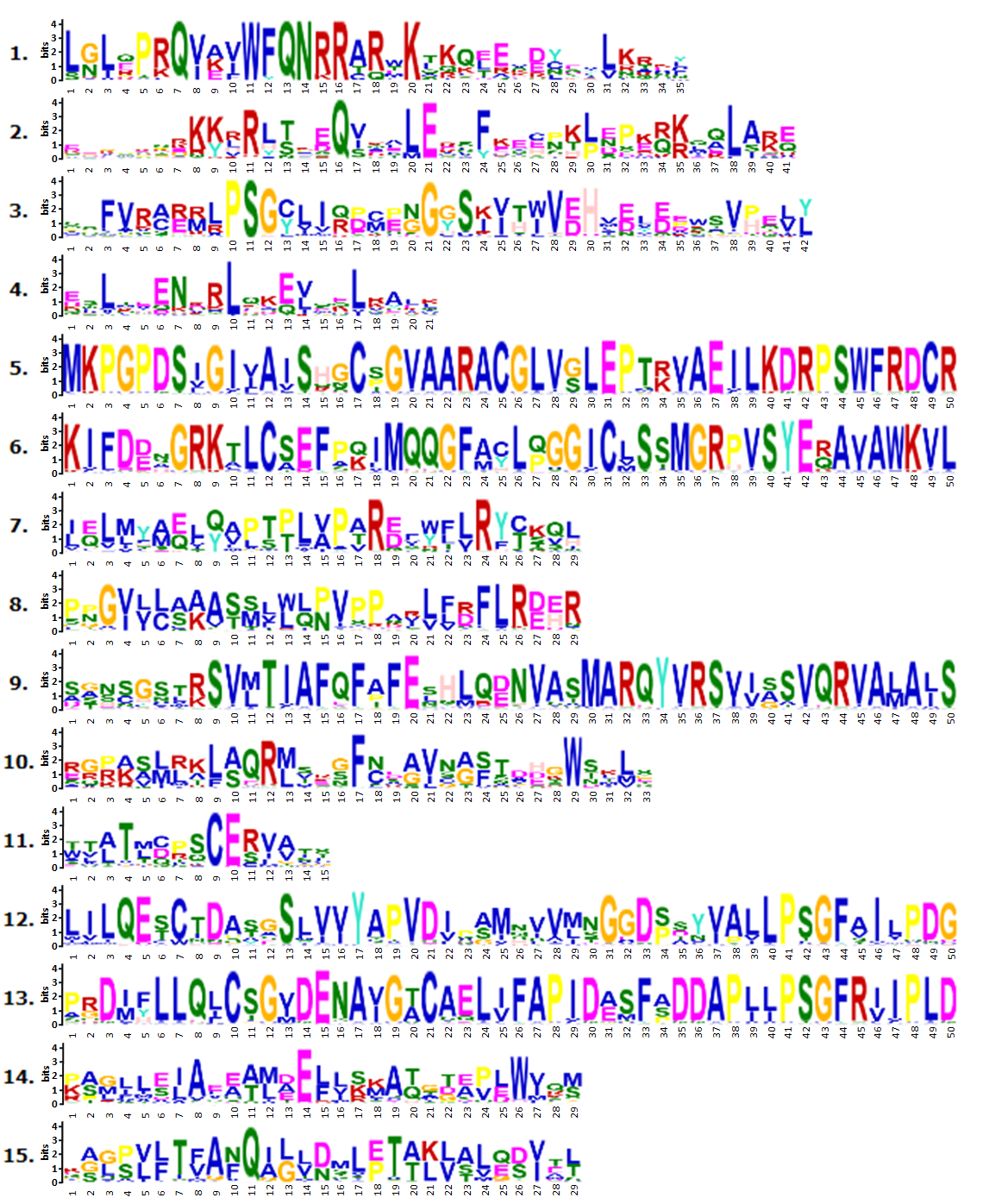

Supplement: S2 Fig — (TIF) [file pone.0173043.s002.tif]

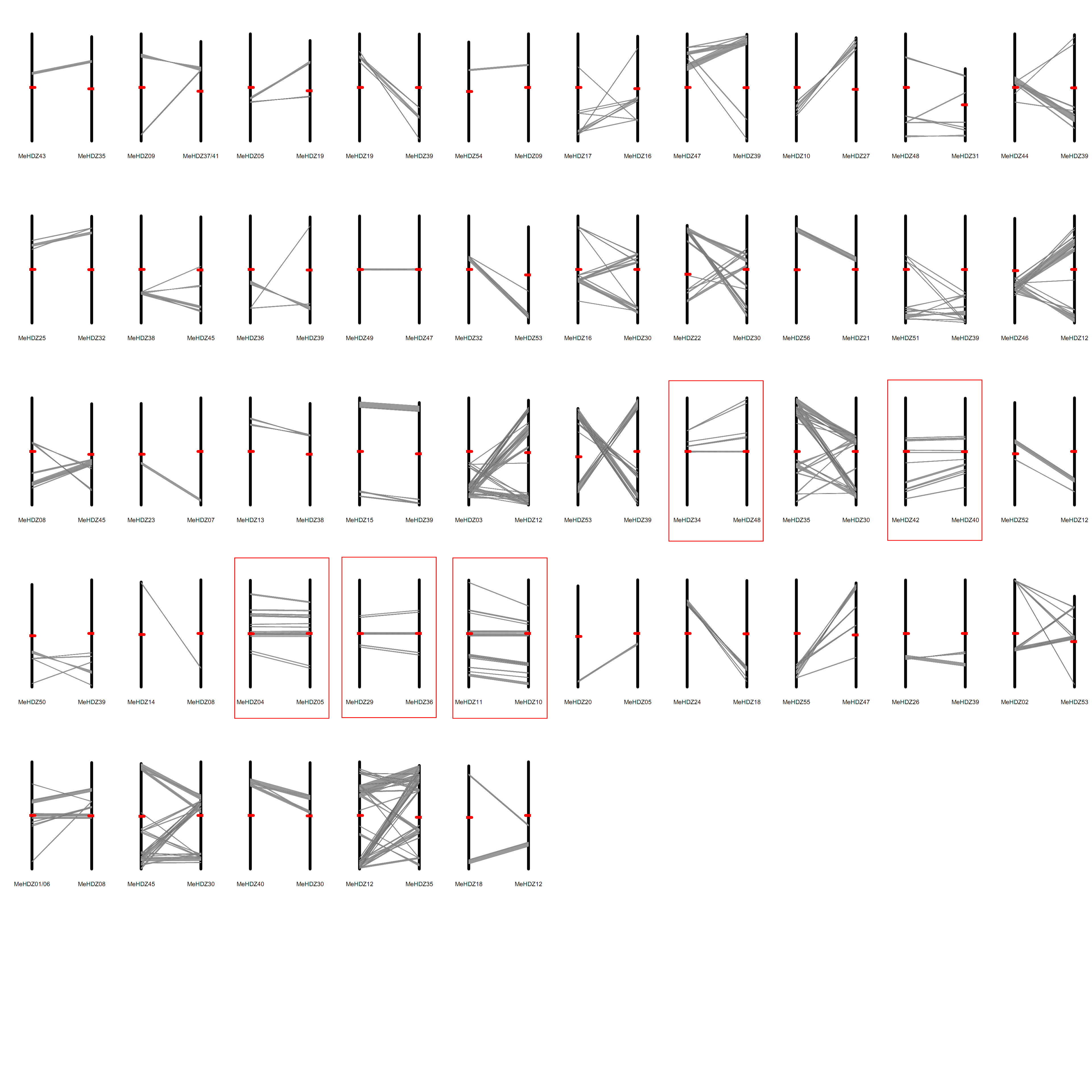

Supplement: S3 Fig — Red dots represented the relative positions of MeHDZ genes indicated at the bottom. Each 50 kb sequences upstream and downstream of the genes were selected to BLASTN against each other, and alignments with length > 200 bp and sequence identity > 85% were chained. Five pairs of MeHDZ genes identified as segmental duplications were marked with red boxes. (TIF) [file pone.0173043.s003.tif]
